# Protein Kinase D Enzymes as Regulators of EMT and Cancer Cell Invasion

**DOI:** 10.3390/jcm5020020

**Published:** 2016-02-03

**Authors:** Nisha Durand, Sahra Borges, Peter Storz

**Affiliations:** Department of Cancer Biology, Mayo Clinic, 4500 San Pablo Road, Jacksonville, FL 32224, USA; durand.nisha@mayo.edu (N.D.); borges.sahra@mayo.edu (S.B.)

**Keywords:** Protein Kinase D, PKD, EMT, migration, cancer

## Abstract

The Protein Kinase D (PKD) isoforms PKD1, PKD2, and PKD3 are effectors of the novel Protein Kinase Cs (nPKCs) and diacylglycerol (DAG). PKDs impact diverse biological processes like protein transport, cell migration, proliferation, epithelial to mesenchymal transition (EMT) and apoptosis. PKDs however, have distinct effects on these functions. While PKD1 blocks EMT and cell migration, PKD2 and PKD3 tend to drive both processes. Given the importance of EMT and cell migration to the initiation and progression of various malignancies, abnormal expression of PKDs has been reported in multiple types of cancers, including breast, pancreatic and prostate cancer. In this review, we discuss how EMT and cell migration are regulated by PKD isoforms and the significance of this regulation in the context of cancer development.

## 1. Introduction

**Figure 1:**
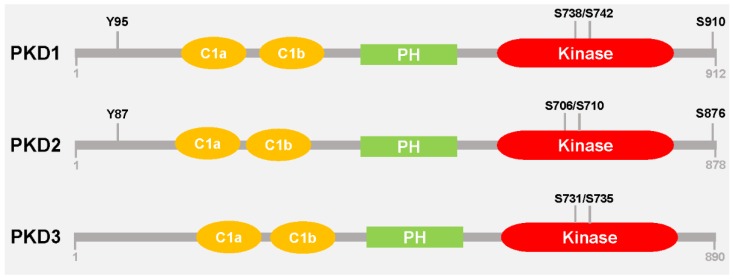
Schematic representation of PKD isoforms. PKD1, PKD2, and PKD3 exhibit high homology with the presence of two cystein-rich domains (C1a, C1b), a pleckstrin homology domain (PH) and a kinase domain in all three isoforms. However, only PKD1 and PKD2, but not PKD3 have a C-terminal autophosphorylation site within a PDZ-binding motif (S910 in PKD1; S876 in PKD2); and an N-terminal tyrosine residue that is phosphorylated by Src (Y95 in PKD1; Y87 in PKD2). The serine residues highlighted in the kinase domain are within the activation loop and are phosphorylated by nPKCs.

Protein Kinase D family members are serine/threonine kinases that function downstream of the novel Protein Kinase Cs and diacylglycerol. The PKD family consists of three isoforms: PKD1/PKCµ, PKD2 and PKD3/PKCν [1]. PKD1 was the first isoform identified [2,3] and is the most extensively studied. PKD family members are characterized by two N-terminal cysteine-rich zinc finger domains (C1a and C1b) which bind DAG and phorbol esters, a central lipid/protein binding pleckstrin homology (PH) domain, and a C-terminal kinase domain (Figure 1) [4]. The C1 domains are thought to play crucial roles in targeting PKD to distinct cellular compartments. For example, recruitment of PKD1 to the trans-Golgi network is mediated by its C1a domain [5], while the nuclear import of PKD1 is facilitated by its C1b domain [6]. On the other hand, deletion or point mutations within the PH domain increases PKD1 basal kinase activity, suggesting that this domain functions in repressing the catalytic activity [7].

In response to stimulation by a variety of biological agents such as DAG, phorbol esters, growth factors and activators of G-proteins, the novel PKCs phosphorylate PKD at two conserved serine residues in its activation loop; in PKD1 Ser^738^ and Ser^742^, in PKD2 Ser^706^ and Ser^710^, and in PKD3 Ser^731^ and Ser^735^ (Figure 1) [4,7,8,9]; leading to activation of the kinase. In addition to activation through the canonical DAG-PKC signaling cascade, increasing evidence suggests that activity can be achieved through several other mechanisms. For example, PKD activation has been shown to occur in response to oxidative stress [10,11,12], binding of G beta-gamma (Gβγ) proteins to the PKD family PH domain at the Golgi [13], as well as caspase 3-mediated proteolytic cleavage [14].

PKDs have been shown to regulate a variety of cellular processes including cell migration [15,16,17], epithelial to mesenchymal transition (EMT) [18,19,20], vesicular transport [21,22,23], stress-induced survival responses [10], angiogenesis [24] and gene transcription [25]. Important physiological roles so far identified are roles in initiating host innate immune responses [26], coordination of the release of insulin from pancreatic cells [27], and increase of cardiomyocyte contractility [28]. In addition, dysregulation of PKD expression or activity has been described to contribute to the development and progression of various malignancies, including breast cancer, pancreatic cancer and prostate cancer [19,29,30,31,32,33,34,35,36,37,38,39,40,41,42,43]. As the founding member of the PKD family, PKD1 has been the focus of most studies. However, more recently, efforts have been geared towards elucidating the functions of PKD2 and PKD3 [34,39,42,44,45,46,47]. For example, Huck *et al.* identified G-protein-coupled receptor kinase-interacting protein 1 (GIT1) as a PKD3 specific substrate [46], and CD8^+^ T cell-dependent immune responses have been shown to be dependent on PKD2 [47]. The results from the aforementioned studies demonstrate that PKD isoforms, dependent on the signaling pathway, exhibit redundant or non-redundant functions. Even though PKD family members have similar modular structures, they do exhibit some structural variability. These structural differences may help account for the distinct functions of PKD isoforms. For example, PKD1 and PKD2, but not PKD3 possess a C-terminal autophosphorylation motif (in PKD1 S^910^ and in PKD2 S^876^) within a PDZ binding motif [48]. In addition, both possess an N-terminal tyrosine phosphorylation motif (in PKD1 Tyr^95^ and in PKD2 Tyr^87^), which is phosphorylated by Src in response to oxidative stress (Figure 1). Src-dependent phosphorylation of PKD1/2 at this residue generates a binding site for PKCδ which facilitates activation loop phosphorylation and ultimately activation of the kinase [12].

The differential expression pattern of PKDs in cancer provides compelling evidence for their non-overlapping functions. For example, PKD1 is highly-expressed in ductal epithelial cells of the normal breast while its expression is downregulated in highly-invasive breast cancers [29,49]. On the other hand, highly-invasive breast cancers are characterized by increased expression of PKD3 [30,33]. Loss of PKD1 and upregulation of PKD3 in invasive breast cancer suggests that in this malignancy PKD1 functions as a tumor suppressor while PKD3 functions as an oncoprotein [15,18,30,33]. Unlike PKD1 and PKD3, the expression pattern of PKD2 remains relatively unchanged during breast cancer progression [30,49]. However, evidence from multiple studies indicates that it also supports breast cancer development by promoting cell migration, proliferation and multi-drug resistance [31,32,50].

Below, we discuss how PKD isoforms contribute to EMT and cell migration, two biological processes relevant for tumor development and progression.

## 2. EMT and Cell Migration

EMT is the process by which a polarized epithelial cell undergoes biochemical changes that allow it to transition to a mesenchymal cell. Epithelial cells undergoing EMT lose their polarity and reorganize their actin cytoskeleton, which enhances their migratory capacity. EMT has been described to occur in three distinct settings; (i) embryonic development; (ii) inflammation and fibrosis; and (iii) invasion and metastasis [51]. In cancer, the EMT process has been associated with the ability of cells to escape from primary epithelial tumors and to colonize novel sites in the body. Interestingly, mesenchymal-to-epithelial transition (MET), the reverse of EMT is also needed for metastases to establish [52]. An EMT is a well-orchestrated process characterized by the activation of specific transcription factors and differential expression of multiple genes [53]. The loss of E-cadherin, a transmembrane protein that mediates cell-cell adhesion in epithelial tissues, has been described as a defining feature of EMT [53,54]. As epithelial tumors progress, they not only lose the expression of E-cadherin but they tend to increase the expression of non-epithelial cadherins such as N-cadherin [54]. A switch from expression of E-cadherin to N-cadherin is a marker for EMT; and during the metastasis process the increased expression of N-cadherin facilitates the formation of cell-cell adhesions that mediate tumor cell invasion [55]. Both, loss of E-cadherin and increased expression of N-cadherin during EMT, are achieved by transcriptional regulation of their genes. For example, the *CDH1* gene which encodes E-cadherin has been shown to be negatively regulated by transcription factors, such as Snail, Slug, ZEB1 and ZEB2 [53]. The regulation of transcription factors involved in EMT is under the control of multiple signaling cascades, including the transforming growth factor-β (TGFβ) pathway [56], as well as the mitogen-activated protein kinase (MAPK) pathway [57]. The repertoire of signaling cascades involved in regulating EMT is continually expanding, and now includes pathways regulated by PKD.

Often, cells that underwent an EMT have a higher potential to migrate. The process of mesenchymal cell migration is characterized by the protrusion of a broad lamellopodium at the leading edge of the cell where polymerization of F-actin filaments and their subsequent contraction generates the force necessary for translocation of the cell body to occur. Polymerization events at the leading edge of the cell are closely associated with the depolymerization of actin filaments towards the rear of the cell [58]. Actin remodeling events in cells are regulated by a diverse group of actin-binding proteins including cofilin, vasodilator-stimulated phosphoprotein (VASP), profilin and the actin-related protein-2/3 (ARP2/3) complex [59,60]. It has been shown that phosphorylation of actin regulatory proteins by PKD isoforms orchestrate migration processes both *in vitro* and *in vivo.*

## 3. PKD1 Maintains the Epithelial Phenotype and Inhibits Cell Migration

**Figure 2:**
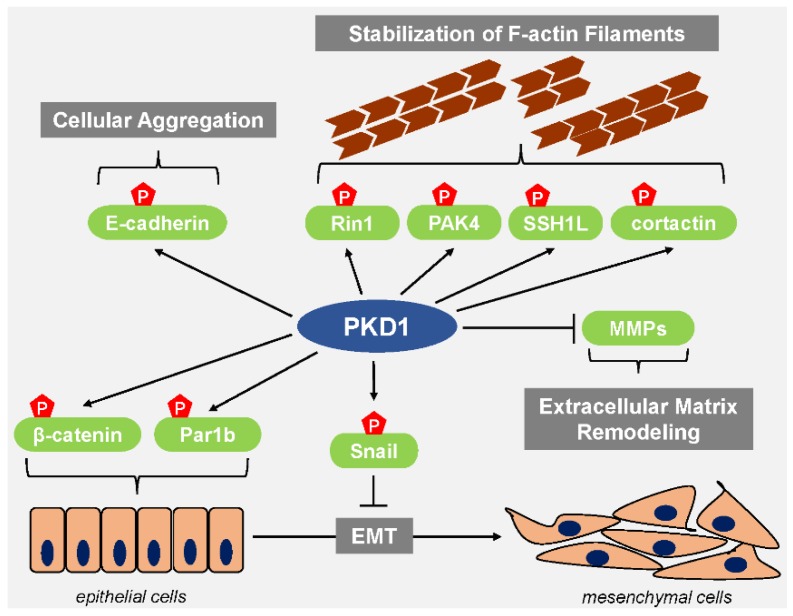
PKD1 blocks EMT and cell migration. PKD1 inhibits EMT by phosphorylation of Snail leading to its inactivation, nuclear export and degradation. Functional consequence is the de-repression of Snail target genes, such as E-cadherin. Phosphorylation of Par1b and β-catenin by PKD1 maintains the epithelial phenotype. PKD1-mediated phosphorylation of E-cadherin causes cellular aggregation and inhibits cell motility. Additional mechanisms of how PKD1 blocks cell migration are by phosphorylation of PAK4, SSH1L, cortactin and RIN1. Net effect of these events is a decrease in actin reorganization and the stabilization of F-actin filaments. In addition, PKD1 prevents expression of MMPs, and, as such, blocks ECM degradation and remodeling.

Data obtained from tissue microarrays (TMAs) shows that PKD1 is expressed in the epithelial ductal tissue of the normal breast [18,49]. Multiple lines of evidence suggest that in this setting PKD1 helps maintain the epithelial phenotype. For example, the transcription factor Snail, which represses E-cadherin expression to induce an EMT is a direct target of PKD1 [18,19]. Phosphorylation of Snail at Ser^11^ by PKD1 inhibits the ability of Snail to repress the expression of E-cadherin and effectively blocks EMT. The mechanism of how Snail transcriptional activity is inhibited by this PKD-mediated phosphorylation has been elucidated in a series of papers. Bastea *et al*. showed that PKD1-mediated phosphorylation of Snail at Ser^11^ reduces its binding affinity for its co-repressor Ajuba and leads to increased expression of E-cadherin [18]. Phosphorylation of Ser^11^ by PKD1 also leads to the interaction between Snail and FBXO11 [20]. FBXO11 is an E3 ubiquitin ligase that targets Snail for ubiquitination and degradation by the proteasome machinery. The phosphorylation of Snail at Ser^11^ also generates a 14-3-3 binding motif and leads to 14-3-3 mediated nuclear export needed for proteasomal degradation [18,61]. As a consequence of downregulation of Snail, E-cadherin is not repressed and EMT is not induced (Figure 2).

An EMT is typically accompanied by the loss of apical-basal polarity [62]. Epithelial cells exhibiting apical-basal polarity have specialized plasma membrane domains which each contain a unique composition of proteins and lipids [63]. Par1b, a serine/threonine kinase associated with the regulation of cell polarity is a direct target of PKD1 [64]. Phosphorylation of Par1b at Ser^400^ by PKD1 generates a 14-3-3 binding motif (Figure 2), and binding of 14-3-3 proteins to Par1b causes it to dissociate from the cell membrane [64]. The PKD1-dependent loss of Par1b from cell membranes to coordinate polarity dynamics in cells is a likely mechanism by which PKD1 helps maintain the epithelial phenotype.

Negative regulation of EMT by PKD1 is also mediated through phosphorylation of β-catenin (Figure 2). β-catenin, along with cadherins and α-catenin are central components of adherens junctions, which mediate cell-cell adhesion. The establishment of epithelial cell layers is dependent on the presence of these adherens junctions [65]. PKD1 interacts with β-catenin and phosphorylates it at two distinct threonine residues, Thr^112^ and Thr^120^. Cells co-expressing wildtype β-catenin and PKD1 showed increased membrane localization of β-catenin, whereas a β-catenin mutant that blocks phosphorylation was localized in the nucleus [66]. This implies that dual phosphorylation of β-catenin by PKD1 facilitates its membrane localization which would in turn enable the establishment of adherens junctions to maintain the epithelial phenotype.

Interestingly, E-cadherin has also been described as a direct target of PKD1 (Figure 2), however, the effect of this phosphorylation does not seem to elicit an effect on EMT but rather enhances cellular aggregation and decreases cell motility [41]. In multiple cancer cell lines, overexpression of constitutively-active or wild type alleles of PKD1 leads to a significant reduction in directed cell migration while an increase in cell migration is observed when PKD1 is knocked down [15,16]. Directed cell migration is a highly coordinated process which requires the reorganization of actin filaments [67]. To initiate such actin remodeling, the actin binding protein cofilin severs actin filaments to generate free barbed ends [59]. Cofilin phosphorylated at Ser^3^ cannot bind actin and is therefore inactive. The levels of phospho-cofilin in cells is regulated by kinases like LIM domain Kinase 1 (LIMK1) and phosphatases like slingshot protein phosphatase 1L (SSH1L) [68]. Continuous phosphorylation and dephosphorylation of cofilin in the lamellipodium is required for cell migration to occur. SSH1L is a direct target of PKD1 (Figure 2), which phosphorylates it at Ser^978^ in its actin-binding motif. As seen for many other PKD1 substrates, this phosphorylation generates a 14-3-3 binding motif, and binding of 14-3-3 to SSH1L promotes their accumulation in the cytoplasm and away from actin filaments at the leading edge of the cell [15,16]. PKD1 also impacts cofilin activity by phosphorylating and activating PAK4 (Figure 2). PAK4 is an upstream kinase for LIMK1 and activation of PAK4/LIMK1 signaling leads to further accumulation of inactive cofilin in cells [17]. Consequently, with increased PKD1 activity, inactive cofilin phosphorylated at Ser^3^ accumulates in cells and free barded ends are not generated, effectively blocking cell migration. In addition to regulation of the cofilin cycle, PKD1 also initiates several other signaling events that negatively-regulate cell migration. PKD1-mediated phosphorylation of RIN1 at Ser^292^ blocks cell migration by stimulating the tyrosine kinase activity of Abl [69]. Phosphorylation of the actin binding protein cortactin at Ser^298^ generates a 14-3-3 binding motif and blocks cell by migration by attenuating Arp complex-driven actin polymerization [70].

PKD1 also regulates the expression of matrix metalloproteinases (MMPs) [49], a protein family that remodels and degrades the extracellular matrix (ECM). Since ECM degradation facilitates the metastasis process, increased expression of MMPs has been reported in multiple types of cancers [71]. Constitutively-active PKD1 decreases expression of several invasion-relevant MMPs including MMP-2, MMP-7, MMP-9 and MMP-10, by so far unknown mechanisms (Figure 2) [49].

## 4. PKD1 in Cancer

Given that PKD1 negatively-regulates EMT and cell migration, reduced expression of PKD1 has been reported in various malignancies [48,72]. In the context of cancer progression, the loss of PKD1 and its effect has been studied most extensively in breast cancer. In a cohort of invasive human breast cancer samples, over 95% of samples had reduced expression of PKD1 when compared to normal breast tissues [49]. This loss of PKD1 in breast cancer is associated with tumor invasiveness. Highly invasive breast cancer cell lines such as MDA-MB-231 and BT-20 do not express PKD1, while minimally invasive breast cancer cell lines such as MCF-7 and normal mammary epithelial cells such as MCF-10A show different levels of PKD1 expression. In invasive breast cancer, the loss of PKD1 is mediated by hypermethylation of the *PRKD1* promoter [29]. Silencing of PKD1 in MCF-7 cells enhances the migratory capacity of these cells. Conversely, overexpression of a constitutively-active PKD1 in MDA-MB-231 cells leads to a significant reduction in cell migration [49]. Results from spheroid invasion assays using MDA-MB-231 cells showed that cells expressing active PKD1 had reduced invasive potential when compared to control cells [49]. The impact of PKD1 expression on the progression of breast cancer has been assessed *in vivo.* Therefore, mice orthotopically implanted with MDA-MB-231 cells, which only express the oncogenic versions PKD2 and PKD3, were treated with the DNA methyltransferase inhibitor decitabine. Mice treated with decitabine had significantly less lung metastases when compared to their saline treated counterparts [29]. Furthermore, restoration of PKD1 expression showed a significant decrease in the quantity of tumor cells expressing MMP-9 as compared to mice implanted with MDA-MB-231 cells expressing PKD1-shRNA (block of decitabine-mediated restoration of PKD1 expression). As discussed above, in the epithelium of the normal breast, the expression of active PKD1 correlates with the phosphorylation of Snail at Ser^11^. Accordingly, the loss of PKD1 in invasive breast cancer is associated with the loss of Snail phosphorylated at Ser^11^ leading to induction of EMT as determined by increased expression of N-cadherin and loss of E-cadherin [18,20].

Reduced expression of PKD1 has been reported in the androgen-independent prostate cancer cell line C4-2 when compared to its parental androgen-dependent LNCaP cells. Moreover, in human prostate cancer progressing to androgen independence, the loss of PKD1 is seen [40]. In the androgen-independent prostate cancer cell line DU145, silencing of PKD1 induces the expression of mesenchymal markers like vimentin and N-cadherin. Conversely, ectopic expression of PKD1 in C4-2 cells induces the expression of epithelial markers like E-cadherin [19]. In a panel of gastric cancer cell lines, the loss of PKD1 mRNA was detected in over 70% of all the cell lines assessed. Reduced expression of PKD1 mRNA was also detected in gastric tumor tissue when compared to adjacent normal tissue. As seen in the case of breast cancer, the loss of PKD1 in gastric cancer occurs due to hypermethylation of the *PRKD1* promoter [73]. Depletion of PKD1 in gastric cancer cells augmented their invasion capability [73]. Loss of PKD1 has also been reported in other solid cancers such as colorectal cancer [74]. Although the downregulation of PKD1 has been demonstrated in multiple solid cancers as a mechanism to increase EMT and cell migration, more comprehensive studies are needed to identify the key mediators driving such signaling.

## 5. Contribution of PKD2 and PKD3 to EMT and Cell Migration

**Figure 3:**
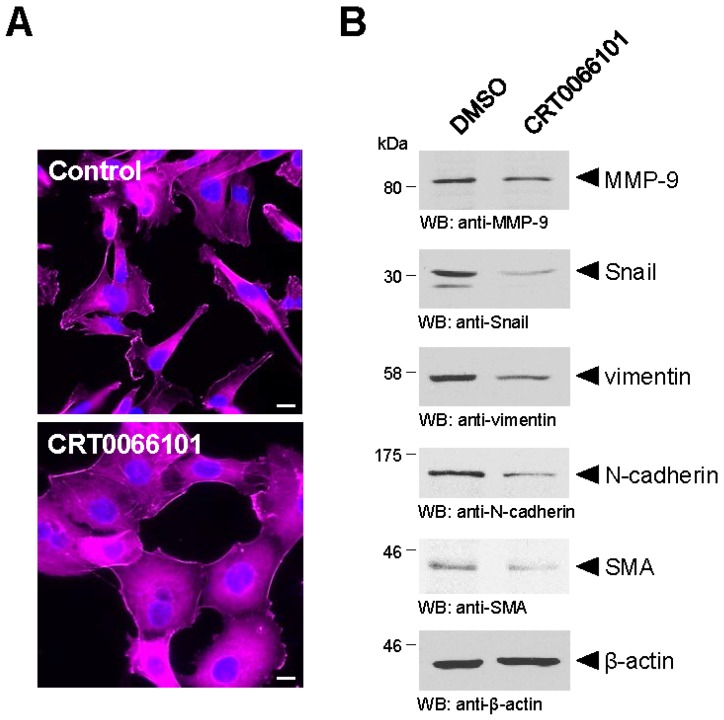
The pan-PKD inhibitor CRT0066101 in PKD2/PKD3-positive cells decreases the expression of markers of EMT. (**A**) MDA-MB-231 cells, which only express PKD2 and PKD3, but not PKD1, were treated with the pan-PKD inhibitor CRT0066101 or control (DMSO). Changes in cell morphology were detected using phalloidin. DAPI staining served as a nuclear marker. The bar represents 10 µm; (**B**) Shown are Western blot analyses of cell lysates of MDA-MB-231 that were treated with CRT0066101 or control (DMSO) for typical markers of EMT. Markers analyzed were MMP-9, Snail, vimentin, N-cadherin, smooth muscle actin (SMA). Probing for β-actin served as a loading control. All detailed methods for above analyses can be found in [30].

The differential expression pattern of PKD2 and PKD3 when compared to PKD1 in breast cancer suggests that the PKD family members do not exhibit the same functions in biological processes like EMT and cell migration [48]. While an inhibitory effect of PKD1 on cell migration and EMT has been the focus of multiple studies, little to nothing is known on the roles of PKD2 and PKD3 in the regulation of EMT. Indirect evidence indicates that PKD2 and PKD3 are potential positive regulators of EMT. This observation was made in experiments conducted using MDA-MB-231 cells, which do not express PKD1, but express both PKD2 and PKD3 [29]. MDA-MB-231 cells treated with the pan-PKD inhibitor CRT0066101 compared to control cells treated with DMSO show a change in morphology (increased spreading of cells) that is indicative for a decrease in motility and EMT (Figure 3A). This is confirmed by analyses for *bona fide* EMT markers like Snail, N-cadherin, matrix metalloproteinase-9 (MMP-9), smooth muscle actin (SMA) and vimentin, which all were reduced in cells in which PKD2/3 were inhibited by CRT0066101 (Figure 3B). However, the relative contribution of PKD2 or PKD3 to processes that regulate EMT remains unclear and needs to be assessed by specific inhibition or knockdown of each isoform.

**Figure 4:**
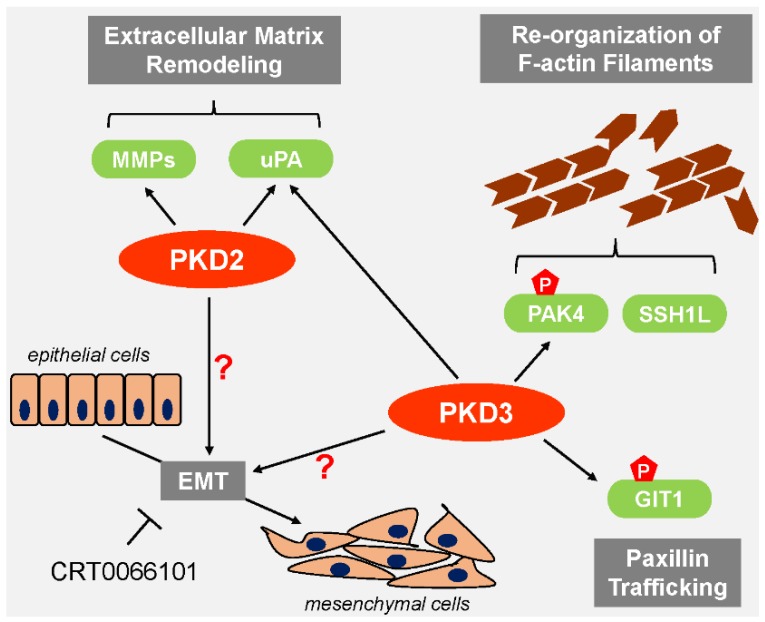
PKD2 and PKD3 support EMT and cell migration. PKD2 and PKD3 form a complex in which basally-active PKD3 stimulates PAK4. Along with activated SSH1L, this leads to the dynamic reorganization of F-actin filaments necessary for cell migration. Both, PKD2 and PKD3 increase the expression and activation of uPA and MMPs augmenting ECM remodeling and enhancing cell migration. PKD3-dependent phosphorylation of GIT1 affects paxillin trafficking and drives cell migration. Use of the pan-PKD inhibitor CRT0066101 in PKD2/PKD3 positive cells with mesenchymal phenotype also demonstrated that these oncogenic versions of PKD may be potential inducers of EMT, but the prospective regulatory molecules in this pathway have not been identified.

Both PKD2 and PKD3 have been implicated in mediating cell migration. For example, silencing of PKD2 in doxorubicin-resistant MCF-7 cells led to a significant reduction in cell migration [50]. Prostate cancer cell migration and invasion are dependent on both PKD2 and PKD3 [43]. However, knockdown of PKD3 when compared to PKD2 had a more pronounced effect on cell migration. As seen for PKD1, PKD2 and PKD3 can impact the cofilin phosphorylation status (Figure 4). Using highly-migratory cells that express PKD2 and PKD3, but not PKD1 as a model system, it was shown that PKD3 can be constitutively-active and that this basal activity is sufficient to stimulate PAK4/LIMK signaling, while SSH1L activity is not affected. This ensures that the cofilin phosphorylation and dephosphorylation cycle at the leading edge is functional and cell migration is maintained [75]. In addition to affecting the cofilin cycle, the stimulating effects of PKD3 on cell migration can also be attributed to its ability to phosphorylate GIT1 [46]. Incorporation of GIT1 into motile, paxillin-positive adhesion complexes is dependent on its phosphorylation at Ser^46^, which is mediated by PKD3 (Figure 4).

## 6. PKD2 and PKD3 in Cancer

Both PKD2 and PKD3 have been associated with the progression and maintenance of various malignances. During breast carcinogenesis, PKD2 levels remain relatively constant, but PKD3 expression is upregulated with increasing aggressiveness [29,30]. For example, triple negative breast cancers (TNBC), the most aggressive forms of breast cancer, have been reported to have elevated levels of PKD3 mRNA [33]. It was also shown that the estrogen receptor can act as a suppressor of *PRKD3* gene expression and that its loss dramatically upregulates PKD3 [30]. Increased levels of PKD3 were detected in human prostate cancer samples when compared to normal prostate tissues. Additionally, there was a strong correlation between PKD3 nuclear localization and increasing tumor grade [39]. In colorectal cancer, PKD2 is the most abundant isoform [74]. PKD2 is also relevant for the development of glioblastoma multiforme (GBM), since high expression of the kinase has been reported in both low-grade and high-grade human gliomas. In human GBM cell lines, PKD2 is constitutively-active and its expression is considerably higher as compared to normal human astrocytes [76].

Both, PKD2 and PKD3 are necessary for the expression of genes associated with metastasis and invasion, such as urokinase-type plasminogen activator (uPA) and MMP-9 (Figure 4) in prostate cancer cells [43]. Like MMP-9, uPA is a protease involved in ECM remodeling and associated with cancer progression [77]. This effect of PKD2 on MMP expression and activity was also demonstrated in pancreatic cancer cells. Overexpression of PKD2 in Panc89 cells led to a robust increase in expression and secretion of MMP-7 and MMP-9 [34].

## 7. PKD as a Potential Therapeutic Target

Across multiple types of cancers, the expression pattern of PKD family members is highly variable. PKDs depending on the tumor type can either promote carcinogenesis or block tumor progression. This differential expression pattern seen in cancer and the tumor-inducing or tumor-inhibiting properties of PKD isoforms can partly be attributed to structural variances and substrate specificity among the different PKD family members. Although currently it is not possible to selectively target each PKD isoform, the unique expression patterns of PKDs in some types of cancer makes them clinically relevant targets. For example, in breast cancer and gastric cancer the loss of PKD1 has been shown to be mediated through hypermethylation of the *PRKD*1 promoter [29,73]. In this setting, the use of DNA methyltransferase inhibitors such as decitabine can be used to reintroduce the expression of PKD1. Decitabine is a Food and Drug Administration (FDA) approved DNA hypomethylating agent that is currently used in the treatment of hematologic malignancies like myelodysplastic syndrome (MDS) [78]. The ability of this drug to alter PKD1 expression has already been evaluated in animal models; using an orthotopic mouse model of breast cancer, Borges *et al.* showed that treatment with decitabine which restored PKD1 expression decreased the ability of breast cancer cells to metastasize to the lungs [29]. An equally efficient strategy in cancers that underwent a switch from expression of PKD1 to the oncogenic versions PKD2/3 is the use of pan-PKD inhibitors like CRT0066101. CRT0066101 is a potent small molecule PKD inhibitor that targets all three PKD isoforms and is orally bioavailable [74]. CRT0066101 has been tested in animal models of breast, pancreatic and colorectal cancer where it was shown to block tumor growth and metastasis to distant organs [30,36,74]. Results from all of these studies indicate that this drug is well tolerated in mice and produced no side effects thus making CRT0066101 an ideal candidate for clinical development. Additionally, the possibility of combining treatment strategies aimed at targeting PKDs in cancer with other chemotherapeutic agents exists. However, before PKD-based therapies can be implemented in the clinic the expression pattern of each PKD isoform has to be determined so that the appropriate treatment regimen can be decided upon [79].

## 8. Summary and Perspectives

There is a growing body of evidence which shows that PKD family members are key regulators of EMT and cell migration. It is well-established that PKD1 through the phosphorylation of Snail and other regulatory molecules contributes to the maintenance of the epithelial phenotype and blocks cell migration. On the other hand, the evidence supporting the pro-oncogenic functions of PKD2 and PKD3 in cancer is convincing, but the precise mechanisms by which these kinases contribute to EMT, regulation of MMPs and eventually cell invasion is still not fully understood. Thus far only limited data is available suggesting that both kinases may be inducers of the EMT process (Figure 3) and further investigation to delineate the exact mechanisms by which they regulate EMT is needed. With regard to cell migration, like EMT, PKD family members do not impact this biological process in the same manner. Though all three PKD isoforms influence the cofilin cycle as well as MMP expression and activity, the outcomes are very different. Due to different targets and level of regulation of the cofilin cycle, PKD1 blocks cell migration while PKD2 and PKD3 drive cell migration. Likewise, PKD1 attenuates MMP expression and activity to keep cell migration processes in check, while PKD2 and PKD3 are positive regulators of MMP activity. Since EMT and cell migration are relevant for tumor development and progression, the expression of PKD family members in multiple types of cancers is deregulated. Studies using cancer cell lines and animal models in which the expression or activity of PKD isoforms have been altered have produced promising results. However, to further enhance the therapeutic prospects of PKDs, additional studies examining the outcome of PKD deregulation in cancer as it relates specifically to EMT and cell migration are required.
